# Dimensionality Reduction and Subspace Clustering in Mixed Reality for Condition Monitoring of High-Dimensional Production Data †

**DOI:** 10.3390/s19183903

**Published:** 2019-09-10

**Authors:** Burkhard Hoppenstedt, Manfred Reichert, Klaus Kammerer, Thomas Probst, Winfried Schlee, Myra Spiliopoulou, Rüdiger Pryss

**Affiliations:** 1 Institute of Databases and Information Systems, University of Ulm, 89081 Ulm, Germany; 2 Department for Psychotherapy and Biopsychosocial Health, Danube University Krems, 3500 Krems an der Donau, Austria; 3 Department of Psychiatry and Psychotherapy, University of Regensburg, 93053 Regensburg, Germany; 4 Faculty of Computer Science, Otto von Guericke University Magdeburg, 39106 Magdeburg, Germany

**Keywords:** immersive analytics, dimensionality reduction, mixed reality, covariance graph, subspace clustering

## Abstract

Visual analytics are becoming increasingly important in the light of big data and related scenarios. Along this trend, the field of immersive analytics has been variously furthered as it is able to provide sophisticated visual data analytics on one hand, while preserving user-friendliness on the other. Furthermore, recent hardware developments such as smart glasses, as well as achievements in virtual-reality applications, have fanned immersive analytic solutions. Notably, such solutions can be very effective when they are applied to high-dimensional datasets. Taking this advantage into account, the work at hand applies immersive analytics to a high-dimensional production dataset to improve the digital support of daily work tasks. More specifically, a mixed-reality implementation is presented that will support manufacturers as well as data scientists to comprehensively analyze machine data. As a particular goal, the prototype will simplify the analysis of manufacturing data through the usage of dimensionality reduction effects. Therefore, five aspects are mainly reported in this paper. First, it is shown how dimensionality reduction effects can be represented by clusters. Second, it is presented how the resulting information loss of the reduction is addressed. Third, the graphical interface of the developed prototype is illustrated as it provides (1) a correlation coefficient graph, (2) a plot for the information loss, and (3) a 3D particle system. In addition, an implemented voice recognition feature of the prototype is shown, which was considered to be being promising to select or deselect data variables users are interested in when analyzing the data. Fourth, based on a machine learning library, it is shown how the prototype reduces computational resources using smart glasses. The main idea is based on a recommendation approach as well as the use of subspace clustering. Fifth, results from a practical setting are presented, in which the prototype was shown to domain experts. The latter reported that such a tool is actually helpful to analyze machine data daily. Moreover, it was reported that such a system can be used to educate machine operators more properly. As a general outcome of this work, the presented approach may constitute a helpful solution for the industry as well as other domains such as medicine.

## 1. Introduction

For the Industrial Internet of Things (IIoT) [1], any kind of insight into the behavior or the status of a machine is essential to (1) quickly react to potential breakdowns or anomalies and (2) technically realize Equipment Maintenance Systems [2]. Investigations how to display the machine behavior properly, in turn, is denoted as condition monitoring. In existing approaches, condition monitoring mainly relies on Key Performance Indicators (KPIs), such as the Overall Equipment Efficiency (OEE). These indicators are usually displayed on company dashboards. Interestingly, recent hardware developments of smart glasses offer new options through a field called immersive analytics, as well as new opportunities for the development of interactive company dashboards. In immersive analytics, new display technologies for analytic reasoning [3] are investigated. As another important observation, due to the increasing number of powerful sensors and context information offered by contemporary IIoT applications, it becomes more and more necessary to perform visual analyses such as those offered by immersive analytics directly at the machine side. Hereby, augmented-reality applications might be especially able to mitigate several challenges when aiming at the visual inspection of data at the machine side. For example, by using augmented-reality applications, users can be supported to recognize spatial contexts of data in an effective manner. A spatial context, in turn, can be stand for an important machine state or behavior and should be therefore quickly recognized by a machine operator.

**Figure 1:**
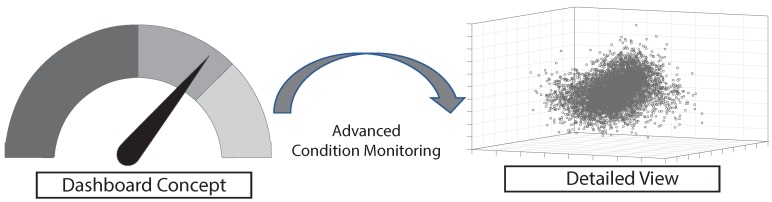
The basic idea of this work is to provide visual, algorithm supported insights into large datasets. As a result, these datasets can be visually inspected to enhance or replace current dashboards features.

Following this, the main contribution of this paper is based on the idea shown in Figure 1: Traditional dashboards (left-hand side of Figure 1; only schematic illustration) will be enriched by more novel features and views that can be obtained by a proper technical setting that incorporates immersive analytics. Using the latter, machine operator will be enabled to quickly recognize spatial contexts of machine data such as the cluster shown in the right-hand side of Figure 1.

**Figure 2:**
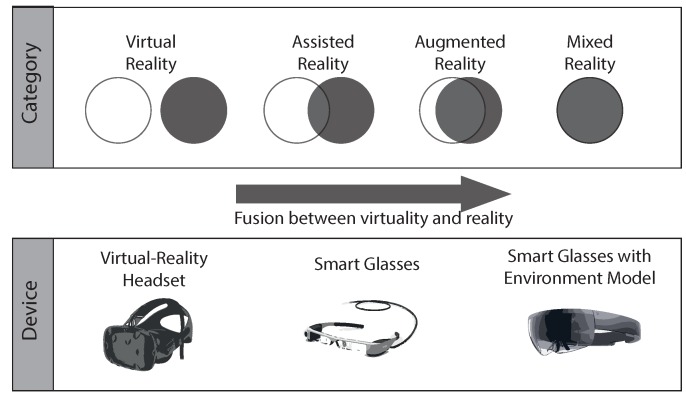
Reality–Virtuality continuum at a glance. The overlap of virtuality and reality increases towards the right-hand side of the figure, the complexity of the necessary devices, respectively.

As augmented-reality applications play an important role in the above-mentioned technical setting, Figure 2 presents a categorization of different approaches in the field of augmented reality for smart glasses. Notably, many other categorizations also exist. However, all categorizations essentially describe the Reality–Virtuality Continuum [4]. In the categorization of the work at hand, the augmented-reality approaches are defined based on the overlap of the boundaries between reality and virtuality. As the first approach, in virtual reality (VR), users are decoupled from the real world by simulating an environment that is comparable to the real word. Usually, this is technically accomplished using a headset. As users have no direct contact with the real world, respective calculations—to relate the user to the real world—are not required. Second, assisted reality (ASR) constitutes an approach in which again headsets are mostly used to augment the real world with information that is not directly in the user’s field of view. That means a user may deliberately change the focus to obtain further information. Consider therefore the following example, if an engineer will repair a machine, a clear field (i.e., without augmented information) of vision is crucially required. On the other, it could be vital for the engineer to be able to check the current machine state in parallel. In this case, assisted reality can be accomplished through a sideways glance to the edge region of the engineer smart glasses, which triggers the display of further information. Third, opposed to assisted reality, augmented reality (AR) shows the relevant information directly in the user’s viewing area. The main difference between assisted and augmented reality constitutes the fact that augmented information is always displayed in the user’s field of view, while in assisted reality they are only shown under certain conditions. Fourth, mixed reality (MR) constitutes another approach, which is used in the work at hand. In this approach of the Reality–Virtuality Continuum, the displayed information is combined with the real world through a concept called spatial mapping, also denoted as 3D reconstruction [5]. When mixed reality will be used, then, first, a room is scanned, usually by the use of specialized sensors (i.e., depth sensors). Based on the sensor results, a model is generated, which serves as the interface between holograms and the real world. Interestingly, mixed reality allows for innovative interaction possibilities in the context of immersive analytics. This is mainly achieved through the deftly placing of diagrams showing additional information. To deal with the basic idea shown in Figure 1, this work uses mixed reality and tackles a set of research question when using this approach of the Reality–Virtuality Continuum for condition monitoring:

- How can mixed reality be used to allow for quick insights into large datasets?
- How can more than three dimensions be visualized in a comprehensible manner?
- How can recommendations be automatically computed for the purpose to visually analyze industrial production systems more properly?

The main goal behind these questions is to investigate whether mixed reality can be helpful to ease the analysis of high-dimensional datasets in condition monitoring. Please note that the presented technical solution was originally developed for a high-dimensional and real-life dataset of tinnitus patients [6]. Here, in this work, the technical solution was extended and adjusted for a production set of additive manufacturing (AM), which is also denoted as industrial 3D-printing [7]. Further note that the complex, multi-dimensional, and hierarchical structure of this use case can be obtained from another work of the authors [8].

**Figure 3:**
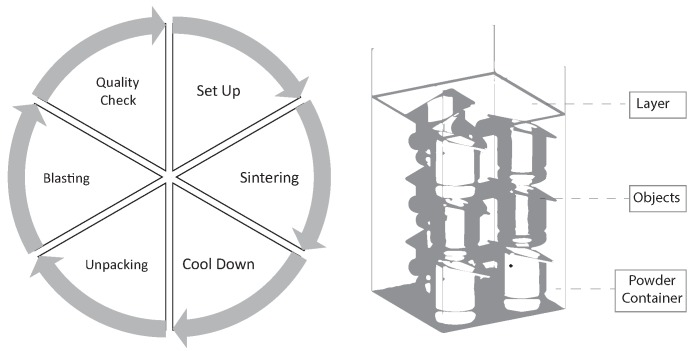
The additive manufacturing process includes several phases in which objects are produced from powder in a layer-wise procedure.

To answer the above raised research questions, we decided to use the Principal Component Analysis (PCA) [9] to cope with the high-dimensional dataset coming from the additive manufacturing scenario we are dealing with (see Figure 3). The PCA, in turn, is often used for classification purposes in the context of high-dimensional datasets. In addition, it is often combined with other approaches such as neural networks. As the PCA aims at the reduction of dimensionality [10], it transfers all gathered values into so-called subdimensions, which allows for displaying 3D plots of various datasets and arbitrary sizes. Such 3D plots are used in the work at hand. On the other, since information can be lost in the transformation process of a PCA, issues emerging pertaining to the dimensionality reduction must be further addressed.

Therefore, this work addresses the issues of dimensionality reduction through the following two measures:

- Identification of clusters in the reduced dataset.
- Recognition of correlations between variables in the reduced dataset.

**Figure 4:**
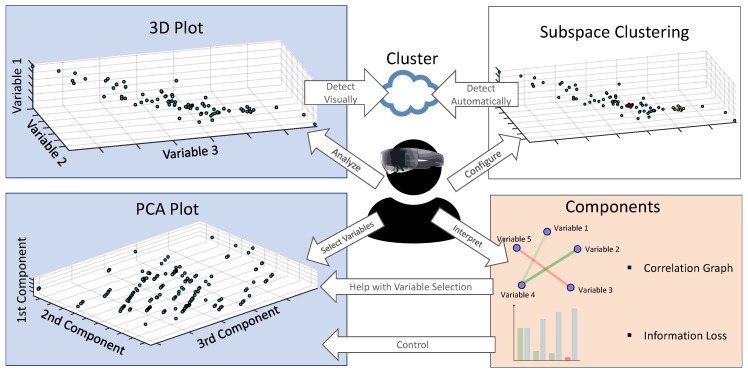
Proposed Approach at a Glance. The 3D plot (**top left**) can be used to display variables and detect clusters. A subspace clustering algorithm (**top right**) reveals automatically detected clusters. With the use of the PCA plot (**bottom left**) and additional components (**bottom right**), dimensionality reduction can be applied and analyzed.

The dimensions we are interested in can be either chosen via the visual inspection of the mentioned 3D plots, or by including an automated feature selection method based on the approach called Subspace Clustering [11]. While the first approach is manually driven, the latter one automatically identifies clusters in high-dimensional datasets. Therefore, Subspace Clustering assumes that relevant dimensions cause multiple clusters in the resulting subspaces. Based on this repeated appearance of clusters, the relevance of dimensions can be automatically selected. In this context, the computational time needs to be evaluated as long as the resulting computational duration might disturb the user when working with the application. To get a better impression of the used ideas and ideas, Figure 4 gives a detailed overview of these features and relates them to the user. Also, importantly, the Microsoft HoloLens is used as the smart glass for the implemented mixed-reality solution proposed by the work at hand.

Furthermore, the approach at hand has been practically evaluated. Therefore, it is shown how the proof of concept has been created using the aforementioned Microsoft *HoloLens* smart glasses as well as the Unity Game Engine [12]. The dataset, which is used to practically evaluate the prototypical implementation, in turn, stems from the following illustrated additive manufacturing scenario of a 3D printer company: In the scenario in question, objects are basically printed in layers using diverse materials. In our use case, metal and plastic are used as the major basis. Using this technique, arbitrary objects can be produced with very filigree structures and advanced production demands, such as cavities, which are generally difficult to produce using subtractive production approaches. Please note that the production process includes several steps. In a first step, the machine operator is setting up a printing job. As several objects are printed at once, the machine operator loads the models of the respective objects to be printed as 3D files—usually constructed by the use computer-aided design (CAD)—into a software and arranges their positions. A safety distance between the objects should also be considered and kept to avoid objects, which eventually might then melt together. After a warm-up phase, the machine sprays new layers of powders in each iteration and a laser is then applied over the contours of the objects. Therefore, the powder melts and sticks together at the heated location, which is denoted as sintering. Next, after finishing the sintering, the powder is removed from the machine and cooled down. Moreover, the machine operator needs to unpack the objects from the block of powder (denoted as powder cake). With a high-pressure cleaner, powder rests are removed from the objects, which is denoted as blasting. Finally, the process is finished by checking the quality of all produced objects in terms of correct dimensions, impurities, and fractions.

Along this process, various data artifacts are generated. The respective data sources, in turn, are

- the geometric structure of the objects,
- the configuration of the machine operator,
- measured values from sensors attached to the machine,
- and the quality report of the printed object.

Due to the high number of possibilities setting up the 3D printing job, it is very difficult to estimate the printing behavior and the achieved quality in the end. Moreover, the progress of the printing job cannot be directly visually inspected. Therefore, an efficient monitoring of the current state is essential and highly demanded.

As the price of the powder is actually very high, production errors should be avoided, and each industrial company has a profound interest in finding correlations and patterns in their production settings. In addition, machine operators are interested in gaining a quick, but understandable insight into the current state of the production. Their knowledge, gained during the production process, needs to be included in further condition monitoring systems. Therefore, the presented approach and developed prototype will support machine operators and data scientists to (a) get meaningful insights into the current production state and (b) be enabled development of hypotheses that can be useful in the improvement of the production processes in general.

The remainder of the paper is structured as follows: Section 2 discusses related work, while Section 3 introduces the mathematical backgrounds for the pursued dimensionality reduction. In Section 4, the developed prototype is presented, in which the dataset, the developed Graphical User Interface (GUI) as well as the backend, and the implemented automated recommendation system are presented. In Section 5, the results of the paper and the threats to validity are discussed. Finally, Section 6 concludes the paper with a summary and an outlook.

## 2. Related Work

To take advantage of 3D-driven approaches in the context of data analytics has been pursued in several fields and scenarios. For example, the authors of [13] showed that 3D visualizations are more useful than 2D representations if a loss of quality will be quantified. More precisely, the authors presented a two-aspect comparison of distance perception, task point classification, and outlier identification. Regarding the first aspect, the authors assessed a visual approach, while for the second approach, they assessed an analytic counterpart. Moreover, in [13], a user study has been conducted, which compared 2D and 3D scenarios on a display. Notably, compared to the work at hand, in [13], no smart glasses have been used. In another study, [14], it was shown that a high degree of physical immersion results in lower interaction times. In this study, the main focus was on scatter plots in immersive environments. Furthermore, in the study presented in [15], the general performance in a 3D space was evaluated. Therefore, the authors compared based on several tests and measurements (e.g., time or identification performance) 2D- versus 3D-based visualizations. In the study of [15], participants had to identify clusters, to determine the dimension of a dataset, and to classify the radial sparseness of data. As with the work at hand, in [16], a prototype was developed and examined for dimensionality reduced scatter plots. Here, the participants had to identify the closest party, party outliers, and the closest deputy in a dataset. The identification was based on user-defined tasks, which were presented in a 3D desktop-based solution as well as an immersive-based 3D solution. Strikingly, the immersive approach showed the best results with respect to the classification accuracy. Compared to this work, in [16], correlations and information were not visualized, nor voice commands were used, and the data points were differently represented. Regarding the latter, in [16], data points were displayed using solid circles and spheres, which we consider as practically not suitable for large datasets such as the one used for this work. Opposed to the discussed works, the authors of [17] favored 2D scatter plots. In their conducted studies, in which users had to compare the class separability of dimensionality reduced datasets, they found out that 3D approaches generated higher interaction costs than their 2D counterparts. The mediation of data analysis concepts, with a focus on abstraction mechanisms, was basically evaluated in an innovative project called Be the Data [18]. Here, participants were embodied by data points, while the floor they stand on represents a 2D projection. Another study aimed on bodily experiences, such as gesturing, body orientation, and distance perception, to support cognitive processes [19]. The Microsoft HoloLens, which was used also in the work at hand, was comprehensively evaluated in [20]. The authors emphasize advantages of working environments in a hands-free manner. On the other, they criticize that a spatial mapping mesh used for their industrial environment is not precise enough. Next, the authors of [21] presented a platform for immersive analytics. Remarkably, the authors describe that they consider an effective data visualization for high-dimensional datasets as the cognitive bottleneck on the path between data and discovery. In our previous research, we demonstrated the advantages of 3D-driven approaches for the analysis of fuel cells [22] as well as quadrocopter flight plans [23], and industrial production parts [24] with the purpose of object recognition. Concerning the research field of subspace clustering, no papers were found that have combined their research with mixed reality. However, an intelligent feature selection using subspace clustering is a common research field. For example, Reference [25] used hybrid approaches of different models and filters to be able to provide recommendations. Here, advantages of algorithms were combined to get a proper meta algorithm. As another example, Reference [26] proposed a greedy feature selection to discover points from the same subset. In Reference [27], in turn, clustering was based on the principal components and on their importance during the clustering process. Finally, the concept of evolutionary algorithms can be used to find clusters in an unsupervised approach [28]. Hereby, the Pareto Front is approximated.

Altogether, the introduced literature shows the potential of immersive analytics, though indicate potential weaknesses in our pursued context. Furthermore, the combination, as shown here, and to the best of our knowledge, cannot be found elsewhere in existing works.

## 3. Fundamentals

### 3.1. Principal Component Analysis

Among others, the Principal Component Analysis (PCA) is an approach, which aims to identify patterns in high-dimensional datasets. Face recognition [29] or image compression [30] are very common use cases, in which a PCA is applied. In essence, the PCA is mainly based on the *covariance* measure of two dimensions x and y. The covariance, in turn, is denoted as:

(1) $$cov\left(X,Y\right)=\frac{\sum_{i=1}^{n}\left(X_{i}-\bar{X}\right)\left(Y_{i}-\bar{Y}\right)}{\left(n-1\right)}.$$

As an important aspect for the interpretation of the covariance, the sign of the result must be considered. If a result value is positive, then *x* and *y* increase together. Conversely, if the value is negative and one dimension increases, then the other one decreases accordingly. Please note that a covariance of zero indicates independent variables *x* and *y*. Further note that if more than two dimensions will be addressed, a covariance matrix is needed, which is denoted as:

(2) $$C^{n\times n}=\left(c_{i,j},c_{i,j}=cov\left(Dim_{i},Dim_{j}\right)\right),$$

where *n* is denoted as the number of dimensions and each entry in the matrix is a result of the calculation shown in Equation (2). Furthermore, the eigenvectors and eigenvalues (Hoffman 1971) of the covariance matrix must be taken into account. Hereby, all eigenvectors of a matrix are perpendicular, whereas the highest eigenvalue is denoted as the principle component. It can be also regarded as the most important axis of a new coordinate system. Each eigenvector, in turn, is identified by a significance, which is also represented by an eigenvalue. The significance, in turn, is the basis for the dimensionality reduction. If components will be removed, then information will be lost. Eigenvectors, which will be not removed, build a feature vector, which is defined as follows:

(3) $$FeatureVector=(eig_{1},eig_{2},eig_{3},\ldots eig_{n}).$$

As the final step, the feature vector will be multiplied with the transposed and mean-adjusted data to receive the final dataset. In summary, the steps, which must be accomplished for a PCA, are as follows:

1. Subtraction of the average across each dimension
2. Calculation of the covariance matrix
3. Calculation of the eigenvectors and eigenvalues of the covariance matrix
4. Definition of the number of components
5. Calculation of the new and reduced dataset

Importantly, through the procedure that excludes eigenvectors, the overall information of the dataset in question is reduced. The information loss, in turn, can be calculated using the percentual significance of the removed components. Generally speaking, correlating dimensions, as expressed by the covariance measure, can be well reduced using a PCA.

### 3.2. Subspace Clustering

The aim of subspace clustering is to find clusters in a high-dimensional dataset. Clusters, in turn, can be used to perform a feature selection. To calculate a cluster, first, it is necessary to define a distance measure [31]. Please note that distance measures become useless in very high-dimensional datasets, which is known as the curse of dimensionality [32]. The used distance measure should be able to handle large and complex datasets. Large datasets often contain irrelevant dimensions, which may confuse the clustering algorithm and are therefore denoted as noise. The difference between traditional feature selection methods and subspace clustering is that the traditional approaches analyze the whole dataset, while subspace clustering is working on multiple, overlapping subspaces. Subspace clustering consists of the two components search method and evaluation criteria. The search method might be a greedy-sequential search, while the evaluation criteria is either a wrapper or a filter model. The wrapper model uses the selection process of a data mining algorithm, while the filter model is based on intrinsic properties of the dataset. Since clustering has no clear definition, there exists no common measure for the clustering quality. It is important to understand that clustering does not guarantee meaningful clusters, but finds interesting patterns for further visual inspections.

In general, there exist two kinds of subspace clustering. The (1) top-down methods start with a clustering on the entire dataset and iteratively searches further on in each sub-cluster, distinguishing between cluster [33] and instance weighting [34]. In contrast to this approach, (2) bottom-up methods find regions with a high-point density in low dimensions and combine them. Therefore, it is essential to define a grid for the region size of clusters. This grid can be either chosen in a static [35] or an adaptive [36] way. In this work, we focus on the static, bottom-up approach CLIQUE [37]. Please note that the latter works in an a priori style [38]. That means, the number of expected clusters is not predefined for CLIQUE. Further note that this algorithm is known to scale up very well with the number of features and is therefore fitting well to our requirements.

#### CLIQUE

**Figure 5:**
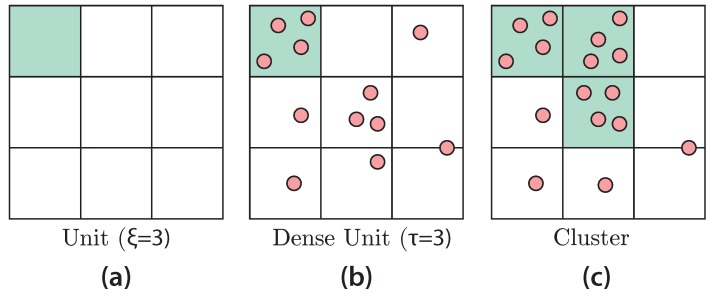
CLIQUE Visual Explanation. (**a**) The user defines his grid resolution and (**b**,**c**) use case dependent thresholds define dense units and cluster definitions.

The basic idea behind the CLIQUE algorithm is to let the user define the degree of resolution in his dataset and how many data points are considered to be a cluster. Based on these parameters, the algorithm can automatically detect clusters in an arbitrary constellation of dimensions in a bottom-up approach. The first step of the CLIQUE algorithm is to subdivide the space into a grid structure (see Figure 5). Hereby, the number of intervals is denoted as ξ. Each resulting tile, or in case of a 3D space a block, is called unit. A unit is denoted as being dense, iff the data points contained exceed a predefined density threshold (τ). The general aim of CLIQUE is to identify the dense n-dimensional units. In case of a 3D space, CLIQUE starts with the three related planes. Therefore, each set of two dimensions is examined and if there are at least two connected dense units, the intersection is recorded as a cluster. When including the next dimension, adjacent clusters are replaced by a joint cluster. Finally, all found clusters are the algorithm’s output.

**Figure 6:**
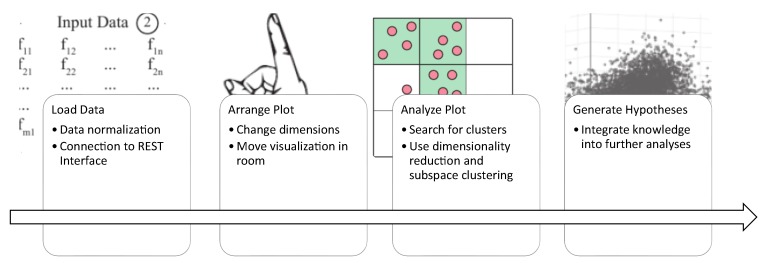
Overall workflow of the implemented prototype and the CLIQUE algorithm.

In what way the CLIQUE algorithm is incorporated into the technical prototype that was developed is shown in Figure 6. The overall workflow starts with a data connection. Afterwards, the user interacts with the data for configuration purposes. Finally, the plot is analyzed, and hypotheses are generated. In the next section, the prototype is technically presented.

## 4. Prototype

The implemented mixed-reality prototype can be basically distinguished to a client and a backend. The client has been implemented based on the Unity Game Engine and the Microsoft HoloLens. If the client side is activated by a user, a hologram is placed in the current room in a static manner. Following this, users can walk around the hologram and evaluate it from different positions.

### 4.1. Dataset

The first version of the prototype was developed based on data from the TrackYourTinnitus project ([39,40,41]). After verifying it for medical purposes, we adjusted the implementation to be able to use it meaningfully for the industrial domain as many aspects are very similar. In particular, the considered additive manufacturing dataset poses similar characteristics. Regarding the considered dataset, it consists of several sub-datasets for each printing job. Each printing job, in turn, contains up to 28 sensors, such as pressure, heating power, gas state as well as frame and laser temperature. The sensor values are collected with a resolution of one value per printed layer. The dataset used for the practical experiment for the work at hand was also cleaned from missing values, which might be caused by sensor failures. To give an overview of the used data, the following table summarizes the cleaned and used dataset for this work.

Please note that the number of layers in each job varies due to the job configuration (see Table 1). Next, each column was normalized to ensure comparability among the dimensions. However, we lose information about the absolute values of each dimension on one hand. On the other, a uniformed representation for three dimensions becomes possible.

A common task for this industrial dataset is to find connections between dimensions and to derive hypotheses, such as *current machine temperature influences production errors*. In this context, seven major requirements concerning the developed application were practically derived from the additive manufacturing scenario:

- **REQ1**: High-dimensional datasets must be displayed in a way that existing clusters can be easily identified.
- **REQ2**: Data representation must be simplified as the application users are not necessarily data science experts.
- **REQ3**: A powerful overview feature must be provided to identify the relation between the datasets dimensions.
- **REQ4**: It must be possible to visualize more than three dimensions as well as to exchange dimensions in the visualization.
- **REQ5**: To enable REQ4 properly, the computational resources must be explicitly considered and well distributed.
- **REQ6**: Precise application feedback and easy input possibilities must be provided to accomplish an understandable data analysis workflow.
- **REQ7**: A recommender system should be provided that supports the decision making while accomplishing the complex process of identifying clusters.

### 4.2. The HoloLens

Importantly for our prototypical implementation, the Microsoft HoloLens incorporates multiple sensors to provide useful user interaction possibilities as well as a proper basis for comprehensive and valuable user feedback (see Table 2). For example, the Inertial Measurement Unit (IMU) offers a combination of accelerometers and gyroscopes, which can improve the visualization of holograms by providing the angular velocity of any head movement [42]. Furthermore, the Microsoft HoloLens offers the use of voice commands, which is promising as they allow for a hands-free interaction principle. Therefore, **REQ6**, among others measures, can be addressed using voice commands. Moreover, a microphone array is provided by the Microsoft HoloLens, which can distinguish between vocal user commands and ambient noises. Moreover, it is possible to identify the direction of external sounds. On top of this, spatial audio can be provided, meaning that the in-app audio comes from different directions, based on the user relative position to virtual objects. This opportunity, in turn, can then be used to guide the focus of a user to relevant diagrams or other important information. Table 2 summarizes the relevant and used technical aspects of the Microsoft HoloLens.

In addition, and important for the prototype, 3D projections can be bounded to real-life objects, which are in the vicinity of a user. That means, only objects from about 60 cm to a few meters can be provided by the Microsoft HoloLens. Also interesting for the work at hand, the HoloLens offers gesture and gaze recognition. However, we only use the tap-to-place-interaction via gestures (see Figure 6).

Some practical aspects that have been emerged when using the Microsoft HoloLens are briefly conveyed. Based on the weight of 579 g, the HoloLens normally requires a longer familiarization phase. Consequently, longer usage periods cannot be recommended when using the HoloLens for the first time. In particular, the unnatural head positioning actions can be exhausting. Although the power consumption of the HoloLens allows for a usage of 2.5 h during intensive use, it is very unlikely that a user will work with the HoloLens that long for an immersive analytics task.

### 4.3. Graphical User Interface

**Figure 7:**
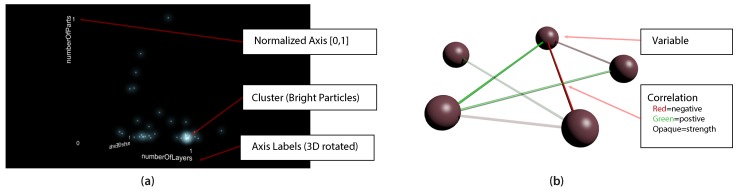
(**a**) Particle-based visualization of additive manufacturing data, where one data point is a print job represented by the variables numberOfLayers, numberOfParts, and numberOfErrors, and (**b**) sample correlation graph to help the user in selecting variables for the visualization.

Figure 7 (left-hand side) introduces the first developed graphical component, which is called the particle system. The increasing brightness for particles in the same position as configured by a shader is addressed here as this effect simplifies the detection of clusters (**REQ2**). More precisely, regions with a high particle density appear brighter than those with only small density contained data points (**REQ1**), which is the basis for the cluster detection. The particle system, in turn, is labeled with the corresponding dimension name on each axis. In addition, the same variable can be plotted on several axes. However, if the axes are overloaded, i.e., more than three variables will be displayed, the visualization will be changed to a dimensionality reduced view (**REQ4**). The latter is implemented based on the PCA and the axes are renamed with respect to the three principal components with the highest significance (see Figure 4). In addition, plot manipulations become possible using voice commands. To be more precise, a hologram can be scaled up or down using the keywords plus and minus. Furthermore, based on the possibilities introduced in Section 4.2, a zoom feature was implemented (i.e., zooming up to 60 cm is provided). Hence, the scaling of the hologram allows for a detailed inspection of data points. As another voice command manipulation feature, the variable assignment to each axis can be edited. Please note that changes in the plots are animated, helping the user to easily recognize state changes. Interestingly, voice commands work only properly until a specific threshold of background noise is exceeded. However, generally speaking, voice commands are experienced as being intuitive and are the basis to satisfy **REQ6**. Finally, note that the prototype for the industrial dataset was validated with experts from the additive manufacturing domain.

All variables that will be displayed are denoted as the variables collection. The items of the variables collection, in turn, are shown beside the particle system as well as in a correlation coefficient graph (see Figure 7, right-hand side, **REQ3**). Regarding the coefficient graph, we have adjusted an existing solution, which was presented in [43]. In particular, the concept of color coding shown in [43] was used and adjusted as follows for the second important graphical component:

- A negative variance is marked with a red edge.
- A positive variance is displayed with a green edge.
- The strength of a variable connection is visualized based on the opacity of each color, where the covariance intervals [0, 1] and [−1, 0] are mapped to the new opacity value in the range [0, 100%].

Although the covariance is used for the PCA calculation, we visualize the correlation as a normalized form of the covariance. The following equation shows how the correlation is denoted.

(4) $$corr\left(X,Y\right)=\frac{cov(X,Y)}{\sigma_{X}\sigma_{Y}}$$

As can be obtained from the equation, there is a dependency between the covariance and correlation. Furthermore, the covariance graph solely marks the strongest edges, while the introduced correlation graph removes irrelevant values by fading them out. With respect to the implemented prototype, users can change the dimensionality reduction by removing variables that do not fit well into the graph. For example, if variables (a) correlate negatively or (b) correlate very weakly, then it is beneficial to remove them.

**Figure 8:**
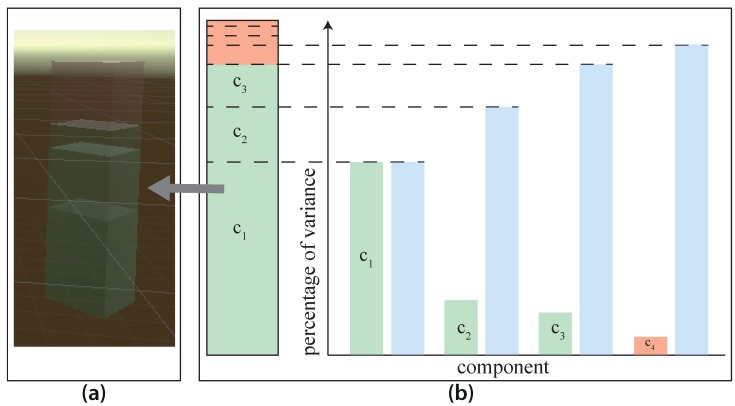
The Information Loss Component, explained by (**a**) the resulting 3D component as a stacked bar, and (**b**) the composition of the stacked bar, which is generated by the variance of the components.

The third and last graphical component focuses on the information loss that is accompanied by the PCA. More specifically, a bar plot shows the percentage of the three most important components for the overall variance. For a better understanding of the bar feature, Figure 8 presents the variance of each component in a stacked bar. Users can recognize the importance of each component based on the transparency. Therefore, the red cube represents the discarded information.

When combining the presented graphical components, their features will enable users to properly work with the dimensionality reduced views. For this purpose, first, emerging challenges when evaluating a covariance matrix are tackled by transforming the latter into the evaluation of a graph. Hence, a quicker visual registration of connections between features becomes possible. Second, the particle system allows for the visualization of high-dimensional datasets and simplifies the detection of clusters through the evaluation of the brightness. Third, stacked bars representing the PCA components variance allow for a quicker evaluation of each component’s importance and the generated information loss.

### 4.4. Backend

**Figure 9:**
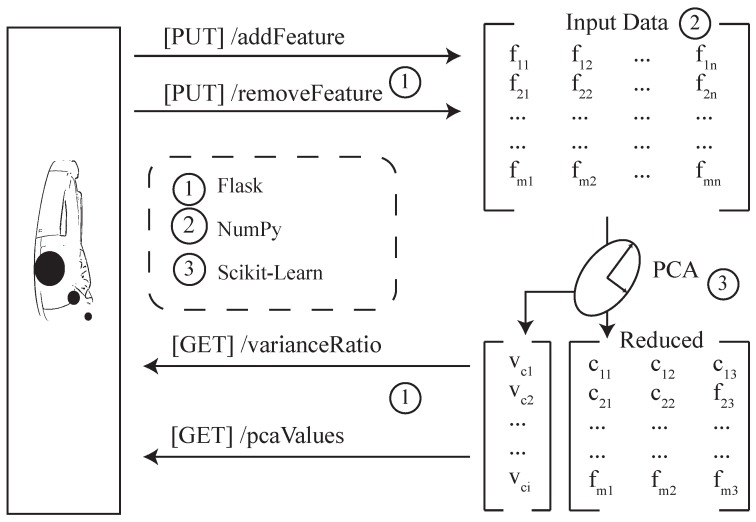
Backend Strategy for providing the PCA-related features. A RESTful-driven architecture is chosen to offer the possibility of working with the infrastructure as a distributed system.

An important strategy of the work at hand is to reduce computational resources required on the used smart glasses (**REQ5**). Consequently, a backend was conceived and implemented based on a python server, which is able to (1) remotely calculate necessary aspects for the smart glasses as well as offers (2) a RESTful API to exchange the data between backend and smart glasses (see Figure 9). Technically, the server backend relies on the web framework Flask [44], which communicates with external applications through the Web Server Gateway Interface (WSGI). As another important technical detail, the implementation of the PCA was done using the free machine learning library scikit-learn [45], as well as the numerical and scientific library NumPy [46]. Finally, the server stores and notices which variables of the dataset have been selected or deselected.

Then, a matrix is calculated as the input for the PCA, which is based on the concrete numbers of selected variables *n* and entries in the dataset *m*. Please note that the PCA is executed for two times, by varying the number of components with the following goals: First, a 3D reduced dataset will be provided, for which only the three components with the highest variances are used. Consequently, the original dataset can now be transformed into the new subspace. Second, the maximal number of components will be provided to illustrate the distribution of their variances. Following this, the prototype can obtain the computed variance ratio vector and the transformed dataset through the provided RESTful API.

### 4.5. Automated Recommendations

In the previous section, the presented interaction with the backend must be done manually. That means, there were no kind of automated recommendations included. In this section, it will be presented how to incorporate the PCA and CLIQUE in a new way to get automatic recommendations for our industrial dataset (**REQ7**) in particular and other similar datasets in general.

#### 4.5.1. Recommendations for Principal Component Analysis

A first automated approach refers to the information loss generated by the PCA. Hereby, the user defines a maximum loss using the implemented voice commands (e.g., maximum loss of 25%). Afterwards, the user defines an initial dimension (e.g., temperature). Now, the developed algorithm shown (see Algorithm 1) tests iteratively the information loss of the PCA by adding a new dimension and keeping the one which exhibits a lesser loss. The algorithm stops when the maximum information loss has been reached.

A similar idea recommends the user to include dimensions based on a change of the relative information loss. For example, if the user included the dimensions temperature, air pressure, and humidity, then, the algorithm can recommend including any other dimension that will not decrease the information loss by more than *x* percent. 

**Algorithm 1:** Recommendation based on information loss. The information Loss is used to remove dimensions from the matrix.

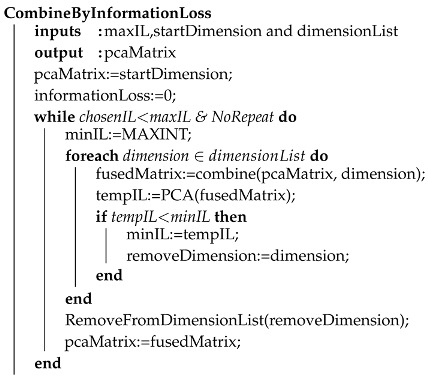



**Figure 10:**
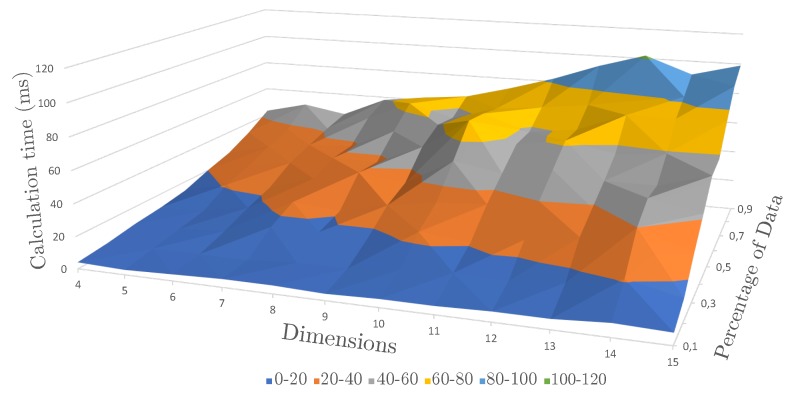
Calculation times for the conducted PCA. In the worst case, the computation takes around 100 ms to calculate the principal components.

As the automated recommendation requires many recalculations of the PCA, we evaluated the computational time for the PCA and its dependencies on the size of the dataset and the number of dimensions (see Figure 10). It is evident that the effect of including more dimensions is decreasing in its numbers relatively to the size of the whole dataset. In general, the achieved (resulting) computational time is low, i.e., a PCA computation for a dataset of 4200 entries with 28 dimensions needs only 57 ms.

#### 4.5.2. Recommendations for Subspace Clustering

**Figure 11:**
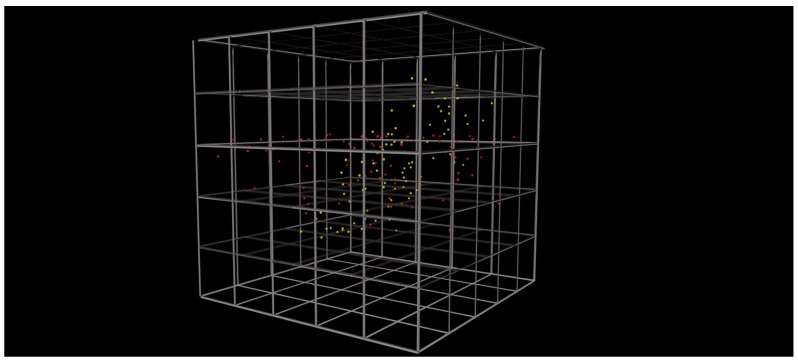
Visualization of two detected clusters (red and yellow) with the predefined subspace clustering grid. Clusters are even detected in overlapping point clouds.

Please note that any clustering result with more than three dimensions cannot be displayed in the HoloLens application. In case of a three-dimensional result, the detected clusters could be visualized as indicated in Figure 11. Moreover, the three-dimensional cube is subdivided into ξ blocks. Please note that this type of visualization is currently not realized in the prototype. Nevertheless, it is possible to receive a speech feedback for the following questions:

- *What is the largest cluster?* This command highlights the clusters that cover the most units and lists all dimensions that are involved.
- *How many clusters does {dimension} generate?* This command searches the list of clusters and counts occurrences of clusters with respect to the selected dimension.
- *What is the densest cluster?* This command compares clusters in terms of data points in the cluster and reveals the one with the maximum number of points.

## 5. Discussion

Strikingly, when practically working with the features presented here, all of them, including the provided command features, were reported to reduce the efforts of a manually applied trial-and-error approach. In general, to be able to actually analyze the data properly, which is recorded in the field of additive manufacturing, we have shown that a prototype or immersive analytics must consider many requirements. Here, we have presented how the features of the prototype can contribute to seven revealed requirements that were identified based on the use of additive manufacturing. In particular, it was shown how the principal component analysis method could be beneficially incorporated. On top of this, it was shown how the implemented features have been combined to allow for reliable, swift, and comprehensive insights into large datasets. Moreover, it was particularly presented in what way users can interact with the prototypical implementation by using voice commands. Furthermore, a state-of-the-art machine learning backend was implemented. Notably, the backend can easily integrate further extensions. We also showed that the backend provides computational resources for the smart glasses in a remote manner with the goal to use the required resources better.

However, limitations and threats to validity need to be discussed. More precisely, it will be shown what has been revealed as the limitations with respect to the practical use of the prototype. In general, although the setting of the smart glass client and the backend enables a powerful computational environment, the installation of the entire technical setting is less intuitive. To be more precise, the client side has technically proven its usefulness as well as its technical feasibility. However, if the whole setting will be used in a large-scale scenario, then the required installation procedure is not appropriate at this stage. Another limitation is the lack of numeric values. That means, for the correlation graph, the stacked bar, and the particle system, no concrete numbers can be used. Consequently, precise data analyzes cannot be accomplished with the current prototype. In addition, the values in the particle system had been normalized, which distorts the impression of the real range of values. Moreover, other subspace clustering methods outperform the selected CLIQUE solution. For example, the successor of CLIQUE, called MAFIA [36], outperform CLIQUE in terms of scalability as well as its feature to provide dynamic grids. The reason CLIQUE has been selected refers to the fact that it has fewer parameters and the static grid offers a better usability for the used smart glass application. Hence, users are probably lesser confused than other solutions in this context. We also observed that the use of the static grid fails in specific cases if clusters are split by the grid and the remaining points are no longer meaningful. To mitigate this, a DBSCAN [47] could be applied. However, the combination of DBSCAN and CLIQUE is not proper. As a more general observation, it could be revealed that the optimization of the subspace clustering parameters is challenging. As a final limitation, the prototype has not been evaluated with respect to the generated mental effort. Therefore, a study could be conducted with the goal to measure the cognitive load when using the prototype practically over a longer period [48].

## 6. Conclusions

In the work at hand, a sophisticated mixed-reality solution has been presented. The aim of this prototype was to reveal better insights on the question how a mixed-reality solution can help to deal with dimensionality reduction effects in the context of immersive analytics. A complex use case—denoted with additive manufacturing—was presented, which stems from the field of Industry 4.0 and for which the presented prototype constitutes a promising solution to support the daily analytics tasks of additive manufacturing workers. Altogether, we consider the three major features that have been implemented, (a) the PCA-specific components (correlation graph and loss function), (b) the machine learning backend, and (c) the implemented recommendation features as being useful for the presented scenario in particular and other scenarios in the field of Industry 4.0 in general. Thus, we consider immersive analytics as being useful for large-scale industrial datasets and that they can be also seen as an important step towards comprehensive analytic solutions in everyday life of engineers and analysts from other domains.

## Figures and Tables

**Table 1:** The Dataset.

Number of Jobs	215
Mean of Number of Layers	411,425
Variance of Number of Layers	57,828
Data Format	XML

**Table 2:** Technical Specifications of the HoloLens.

Inertial Measurement Unit (IMU)	1
Environmental Recognition Camera	4
Depth Sensor	1
RGB Camera	2MP*1
Mixed-Reality Capture	1
Microphone	4(2*2)
Ambient Light Sensor	1
